# Use of robot technology in passive mobilization of acute hospitalized geriatric medicine patients: a pilot test and feasibility study

**DOI:** 10.1186/s40814-019-0545-z

**Published:** 2020-01-06

**Authors:** AS. Bertelsen, A. Storm, L. Minet, J. Ryg

**Affiliations:** 1 Department of Geriatric Medicine, Odense University Hospital, J. B. Winsløwsvej 4, 5000 Odense, Denmark; 2 Department of Rehabilitation, Odense University Hospital, Odense, Denmark; 3 Department of Clinical Research, University of Southern Denmark, Odense, Denmark; 4 Department of Health Sciences, UCL University College, Odense, Denmark

## Abstract

**Background:**

Along with an aging population, the field of robot technology in rehabilitation is expanding. As new technologies develop, it is important to test these clinically before implementation. To assess the possibilities of undertaking a future randomized controlled trial (RCT), the aim of this study was to pilot test and investigate the feasibility of a newly developed passive mobilization robot device in geriatric medicine patients.

**Methods:**

We used a robot to perform passive mobilization for all recruited patients while they were lying in bed. Inclusion criteria include the following: ≥ 65 years of age, able to walk before hospitalization, and not capable of walking > 2 m at the first day of hospitalization. Exclusion criteria include the following: known moderate/severe dementia, unstable fractures (back, pelvis, or legs), high intracranial pressure, pressure ulcers/risk of developing pressure ulcers due to fragile skin, positive Confusion and Assessment Method (CAM) score, not able to understand Danish, and medical instability. A mixed-methods approach, including structured interviews for patients and relatives, questionnaires and semi-structured interviews for the staff, and observations in the clinic were used as data collection methods. A 6-week pilot test preceded the feasibility study to test study design, safety, interview guide, and setting, and to become familiar with the robot.

**Results:**

The pilot test included 13 patients, made the staff confident in the use of the robot, and led to the correction of the interview guide. In the feasibility study, 177 patients were screened, 14 patients (four men, nine women) included, and 13 completed the intervention (median [IQR] age 86 [82–92] years). Overall, the robot was easy to use during passive mobilization and fully accepted by patients and relatives. Staff, however, found the robot difficult to maneuver. No adverse events were reported.

**Conclusions:**

Use of robot technology in passive mobilization of older patients was feasible and well accepted by patients, relatives, and staff. Technical and workflow-related issues, as well as the robot not performing active mobilization, affects the launch of a RCT and thereby its implementation in geriatric medicine patients.

## Background

The population of older people in the industrialized world is increasing [1] leading to a growing number of older individuals with multiple age-related comorbidities and functional disabilities [2]. Worldwide, the aging population is therefore expected to become a future challenge for health care systems [1].

Acute hospitalization is often accompanied by a reduced level of physical activity, and studies show that older patients spend most of their time lying passively in bed [3–5]. Reduced mobility during hospitalization is an important risk factor for adverse in-hospital outcomes and an independent predictor of poor hospital outcomes at discharge, specifically decline in activities of daily living, institutionalization, and increased mortality [3]. Geriatric medicine patients are characterized by comorbidity, polypharmacy, and functional disabilities before hospitalization [6] and have increased risk of further functional decline and increased dependency after acute illness and hospitalization [7, 8]. This makes mobilization of older hospitalized adults an important issue to address in acute rehabilitation research.

The field of robot technology in rehabilitation is expanding with an increase in new devices and technologies emerging each year [9]. The costs of therapist time in healthcare can limit the amount of therapy that is available to the patients due to the growing number of older individuals who need acute rehabilitation. Robots have the potential to increase the amount of therapy received by an individual patient [10], but knowledge on the effect of introducing more robot technologies is lacking. As the new technology develops, it is important to provide evidence-based research before implementing new robots into clinical practice [9]. However, recruitment to randomized controlled trials (RCTs) can be challenging, as many trials fail to reach their planned sample size within the originally expected trial timescale or trial funding might run out [11, 12]. Therefore, an advisable approach is to perform pilot and feasibility studies to determine whether an intervention is appropriate for further testing in pilot and full-scale RCTs, and enable researchers to assess whether or not the ideas and findings are relevant [13].

In order to assess the possibility of undertaking a future RCT, the aim of this study was to pilot test and investigate the feasibility of a newly developed passive mobilization robot device (ROBERT®) in geriatric medicine patients with limited mobility. The objectives of the study were to explore (1) the mobilization sessions (time consumption, number of completers, and adverse events), (2) the management (work environment, workflow, and technical challenges and security), and (3) the perception (experience) of the robot from the view of the patients, relatives, and hospital staff.

## Method

This study is reported using the CONSORT 2010 guidelines for reporting a pilot or feasibility study [14].

### Theoretical framework and study design

To explore the feasibility of the passive mobilization robot, a pragmatic research approach was used [15] and the intervention was given to all participants. To test our feasibility study design, inclusion and exclusion criteria, screening procedure, interview guide, and setting, and to become familiar with the robot, the feasibility study was preceded by a pilot test.

### Participants

We included patients ≥ 65 years of age who were able to walk with or without walking aids before the hospital admission, but not capable of walking with or without walking aid more than 2 m at the first day of hospitalization. All patients were screened systematically during the inclusion period. We excluded patients with known moderate or severe dementia; unstable fractures in the back, pelvis, or legs; high intracranial pressure; pressure ulcers or risk of developing pressure ulcers due to fragile skin; patients with medical instability; patients who were not able to understand Danish; and a positive Confusion and Assessment Method (CAM) score [16, 17]. CAM score was used to identify a patient with potential delirium. Using local standard procedures, a positive CAM score was given in case of an acute onset or fluctuating course, inattention, and disorganized thinking or altered level of consciousness. Furthermore, we excluded patients if the healthcare professional assessed that the patient was not suitable for mobilization sessions with the robot or the patient themselves did not want to be trained by the robot. A clinically experienced physiotherapist was responsible for the recruitment.

### Study setting

The study took place at the Department of Geriatric Medicine, Odense University Hospital, Denmark. The pilot test was performed from September 5, 2018, to October 24, 2018, and the feasibility study from November 6, 2018, to December 12, 2018.

A study board was established prior to study initiation. The board included the head consultant and head nurse of the Department of Geriatric Medicine and the authors of this article: ASB, Master of Science in Public Health; AS, physiotherapist and Master of Health Science; LM, PhD physiotherapist and Head of Rehabilitation Research Unit; and JR, PhD consultant and Head of Geriatric Research unit.

The robot was developed by Life Science Robotics ApS, Aalborg, Denmark.

### Intervention

**Fig. 1:**
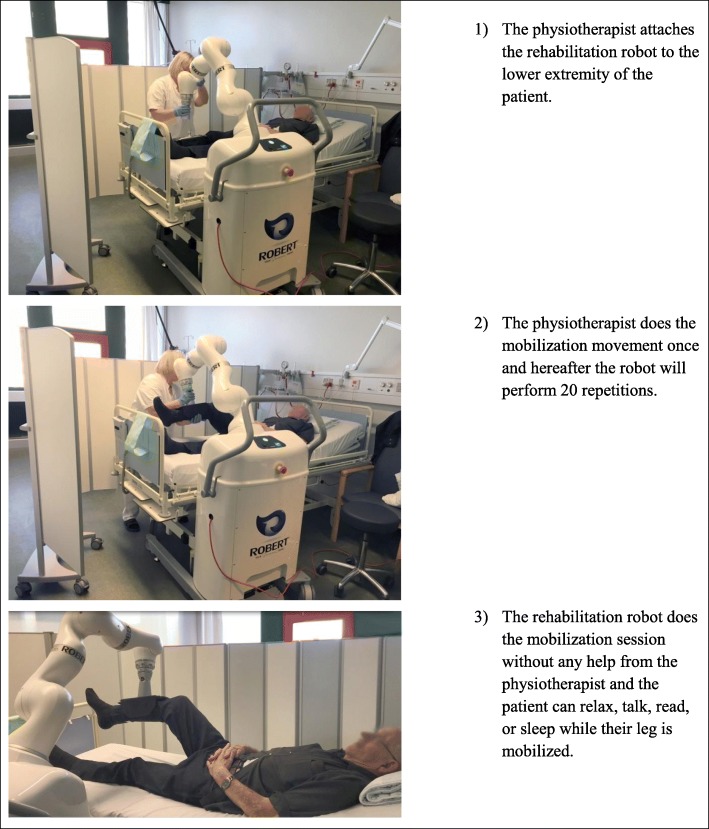
Picture of the passive mobilization robot ROBERT®

The mobilization was performed using a newly developed robot (ROBERT®) which consists of a body on a wheelbase, a robot arm, and a brace with a velcro-sheet that is attached to the lower extremity of the patient (Fig. 1). The robot is provided with a handle to maneuver the device around. When the leg of the patient is attached to the robot, the device is able to perform different passive mobilization exercises designed uniquely by the physiotherapist for each specific patient. The physiotherapist does the movement once, and hereafter, the robot will do 20 repetitions by itself and without any support from the physiotherapist. This enables the physiotherapist to perform other tasks and see other patients, while the robot performs the repetitions by itself. If the patient moves his/her leg against the movement of the robot, it will automatically stop further movement for security reasons. If, for example, the patient has a cramp in their leg, the robot stops in order to prevent pain or injury.

An experienced physiotherapist, who was trained in the management and use of ROBERT® prior to the study, managed the robot daily. A second physiotherapist was also trained in order to step in in case of the absence of the primary physiotherapist. However, this was not needed. The physiotherapists in the department would provide usual care to all patients during the study period.

### Outcomes and data collection

The outcome measurements of this feasibility study focused on (1) the mobilization sessions: time consumption, number of completers, and adverse events; (2) the management: work environment, workflow, and technical challenges and security; and (3) the perception: the experience of the robot from the view of the patients, relatives, and hospital staff (Table 1).

**Table 1:** Objectives, descriptions, study questions, outcome measure, and data sources

Objectives	Description	Study questions	Outcome measures	Data sources
Mobilization session	The use of the robot in the geriatric department	To what extent can the robot be used in its current design?	Time consumption, number of training sessions and completers, and adverse events	Questionnaires and semi-structured interview
Management	How the robot was managed and adapted in the geriatric department	To what extent can the robot be successfully implemented in a geriatric department? Is there a need to change or adapt the robot for the environment?	Working environment, workflow, technical challenges, and security	Questionnaires, semi-structured interview, and observation
Perception	How the geriatric patients, their relatives, and the staff react to the robot	To what extent is the robot suitable to be implemented in a geriatric department?	Perceived acceptability	Structured interviews, semi-structured interview, and observation

#### Structured interviews for patients and relatives

Structured interviews containing pre-planned questions were used to gather data from all patients. This method ensured that all informants in the study were asked the same questions in the same order [18]. The structured interviews were used to obtain information about the patient’s perception and experiences about having passive mobilization performed by the robot. The questions were developed through literature search, experiences from clinical practice, and discussion among authors (Additional file 1: Table S1). To achieve knowledge from the perspective of the relatives, relatives of two patients were also invited to a structured interview. Field notes were made during all interviews if patients and relatives elaborated on their responses. A single interviewer trained in qualitative research conducted the interviews within a week after the patient had finished the mobilization sessions with the robot. The interviews took place in the patient hospital room or by telephone if the patient had been discharged before an interview was possible. To validate data from the interviews, the interviewer performed member checking at the end of every interview. This entailed repeating the key elements of what the participant had told, to secure that the information was gathered in the right way and thoroughly understood [19, 20].

#### Questionnaires and semi-structured interview for the staff using the mobilization robot

Questionnaires were used to gather data from the physiotherapist. The questions were developed through literature search, experiences from clinical practice, and discussion among authors. They contained questions regarding time consumption, technical challenges and security, work environment, workflow, and the reactions of the patients to the mobilization sessions. The physiotherapist filled out a questionnaire after every session. A semi-structured interview was used to gather data in order to explore the physiotherapists’ perception of the daily use of the robot (Additional file 2: Table S2). Furthermore, the physiotherapist was given a scheme to fill out in case of any adverse events.

#### Observation of clinical practice in the use of the mobilization robot

With inspiration from field research, observation was conducted to discover and elucidate possible challenges related to the use of the robot in a real-life setting at the department [21]. Routines, workflow, and mobilization sessions were observed in order to gain insight in the setting in which the robot was implemented.

### Statistics and analysis

The data was analyzed using descriptive statistics for all variables in questionnaires and for the questions in the structured interviews. Due to the aim of the present study, no power calculation was performed. We aimed to include at least ten patients by convenience sampling. By applying the concept and tool, information power, we reflected systematically on sample size and thereby agreed that a small sample size was appropriate for our study [22].

Data from the verbatim transcribed semi-structured interview and observation notes were analyzed using a deductive [21] thematic analysis approach to reveal issues of importance to the feasibility study [23]. All coding and analysis for the semi-structured interview was performed systematically using the software QSR NVIVO Pro 11. The main author did all primary statistics and analysis. Subsequently, the authors discussed these and agreement on results was obtained.

## Results

### Pilot test

During the pilot test, 91 patients were admitted, 86 patients were screened, and 13 patients were included and had a least one mobilization session with ROBERT®. Of these, ten patients were interviewed. The screening procedure was practiced and inclusion criteria tested, as well as the procedure for interviewing the patients. Furthermore, the pilot test made the staff confident about the use of the robot. It was revealed that it was most practical for the physiotherapist to place the robot at one side of the bed and mobilize both legs from this side due to lack of space in the hospital room, time spent, and difficulties moving both furniture and the robot. Furthermore, the pilot test showed that the study design, screening, inclusion, and intervention worked as intended. One question in the interview guide was deleted as it added no new information. No adverse effects were recorded. All members of the study board agreed that it was safe for both patients and staff to proceed with the feasibility study.

### Feasibility study

**Fig. 2:**
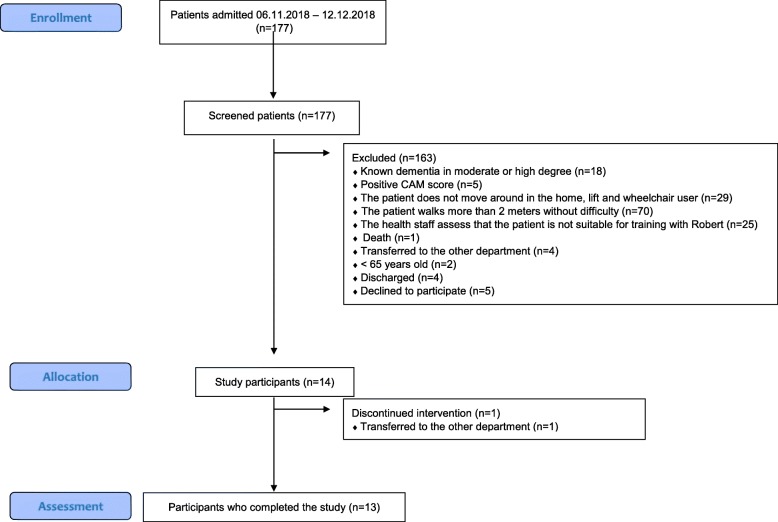
Flowchart of study participants in the feasibility study period 06.11.2018 to 12.12.2018. Inspired by The CONSORT 2010 Flow Diagram

A total of 177 patients were admitted during the period of the feasibility study. All patients were screened, and 14 (four men, nine women) were included (Fig. 2). One patient had to discontinue the intervention due to transfer to another hospital department, and 13 patients (age [median (IQR)] 86 (82–92) years) completed their mobilization sessions.

Twelve patients were interviewed within 1 day after their last mobilization session; ten interviews were held in the hospital room, one by phone, and one was held at a rehabilitation center to which the patient had been discharged. One patient was not interviewed due to decline of cognitive condition. Two relatives were interviewed in the patient hospital room with the patient sitting next to them.

The following sections describe the three objectives of the feasibility study (Table 1).

### The passive mobilization sessions

A total of 10 (10–15) min [median (IQR)] was used to prepare the robot for use. This included bringing the robot to the patient in the hospital room, moving furniture if this was in the way, putting up screens so the mobilization sessions were not visible to everyone passing by (there could be up to 4 patients in the same hospital room), and doing the start-up calibration of the robot. Each participant had 2 (2–3) [median (IQR)] mobilization sessions with the robot each lasting 25 (20–35) min [median (IQR)].

Regarding the quality of the passive mobilization, the physiotherapist expressed in the semi-structured interview that the robot met the expectations:

It is brilliant. It does exactly as it is told (physiotherapist)

It was also emphasized that performing passive mobilization is a heavy task, which can lead to work related pain and injuries. If performing passive mobilization is a big part of the everyday work, the physiotherapist recommended that it would be wise to use the robot:

Physically, the robot clearly spares the back of the physiotherapist (physiotherapist)

Furthermore, the questionnaires filled in by the physiotherapist revealed that the robot was safe to use for both the patients and the physiotherapist during the mobilization sessions. Only one patient felt unsafe during a single session. In 76.5% of all mobilization sessions, the patients did not express any pain or discomfort at any time. In the cases (8/34) where the patient had expressed pain or discomfort, it was caused by a general feeling of soreness in the patient’s leg or the velcro-sheet being too tight around the patient’s leg, which was then immediately and easy loosened.

During the interview, the physiotherapist expressed that it would not be safe for the patient to do exercise with the robot without a physiotherapist being next to the patient in order to provide a safe mobilization session. If the patient were to be left alone in the hospital room while doing exercises with the robot, the physiotherapist pointed out that a stop button would need to be provided for the patient:

One cannot leave the patient without them having a button so they can stop the machine! (physiotherapist)

No adverse events were reported during the study.

#### The management

From the questionnaires and interviews, the robot was reported to be easy to use and to clean. In almost all mobilization sessions, the physiotherapist reported that the robot was easy to apply to the patients’ legs (88.2%) and that the robot did not lack any technical functions (97.1%). When the robot was placed at the opposite side of the mobilized leg, the arm was a little short, and as a general supplementary comment in the questionnaires, it was stated that a longer arm on the robot was desired. Furthermore, it was reported that a display on the robot was missing, as it could be convenient to see the number of repetitions still to come.

In every questionnaire answered by the physiotherapist, it was reported that the robot did not work satisfactorily in the physical environment of the department as it was difficult to maneuver around the bed and other furniture, as well as being difficult to transport to the patient in the hospital room due to its size and weight. Observation of workflow and routines also showed that it took extra time for the physiotherapist to make room for the robot, and even then, it still felt crowded to be in the hospital room with the robot. The physiotherapist pointed out that using the robot was more time consuming than doing passive mobilization manually:

It takes 10 minutes extra to use the robot compared to passive mobilization without the robot. Because I have to move furniture to make room for the robot (physiotherapist)

It was also pointed out in interviews and noticed in the observation that the robot was noisy. The cooler inside the robot was loud and for the physiotherapist an annoying element. However, the patients were not bothered by the sound of the cooler.

#### The perception

##### Structured interviews with patients and relatives

None of the patients had ever tried a device similar to this mobilization robot. Ten of the 12 interviewed patients reported that it was good to do passive mobilization with the robot, while two stated that it was neither nor:

I was very happy about it. I can highly recommend it. It was nice that my legs were being activated, when all I could do was to lie in bed (DF)

It was relaxing. I slept while my leg was being trained (FF)

It just wasn’t for me (CM)

As the above quotes show, the patients were very fond of the robot and a patient furthermore expressed that she was motivated by the mobilization sessions with the robot, which gave her the feeling of being active and on the way to get physically and mentally better.

All the interviewed patients reported that they felt safe while exercising with the robot:

I felt completely safe. After trying it a few times I would also be comfortable even though the physiotherapist was not standing by my side the whole time (DF)

Nine patients had not experienced any unpleasantness related to the robot, two reported that the training had been unpleasant and one reported neither nor. However, the two patients who reported an unpleasant experience also stated that they felt pain before the session and in general.

I already have a lot of pain. I was tired after the workouts (GF)

On the contrary, this patient also pointed out that she missed the sessions with the robot in the weekends where the sessions were on hold.

All patients reported that they had received all necessary information and they had no doubt about anything related to the robot.

Eleven patients stated that they would say yes to do mobilization with the robot if they were admitted again. Furthermore, one patient expressed that the passive mobilization had been good for her legs:

I was calmer in the legs afterwards. They benefit from being activated (DF)

The interviewed relatives (a wife and a daughter) had both attended at least one mobilization session. Both reported that it had been a good experience. They had felt safe to see their loved one doing passive mobilization with the robot and had not experienced anything unpleasant:

My mother thought it was relaxing (relative to FF)

##### Semi-structured interview with hospital staff

The interview with the physiotherapist revealed perspectives about the use of a robot for geriatric medicine patients and about their need for passive mobilization in rehabilitation.

It was the perception of the physiotherapist that the patients liked the robot very much. The physiotherapist uttered in the semi-structured interview that most patients agreed to participate in the study as soon as they had information about the robot:

The robot is easy to “sell” to the patients. They like to hear that they can lie down in the bed and relax, and at the same time exercise their legs. This information makes them say yes to participate (physiotherapist)

However, regarding the likelihood of the robot being implemented at a geriatric medicine department, the interview revealed that geriatric medicine patients might not be a suitable target group for the robot in its current design. The physiotherapist pointed out that the geriatric medicine patients in the study period seemed more in need of active mobilization rather than passive mobilization. Although the patients matched the inclusion criteria, the condition of the patients improved quickly and the physiotherapist therefore assessed that active mobilization would be more beneficial. Due to this assessment, it was a request from the physiotherapist that the robot would be able to perform both active mobilization along with passive mobilization:

Our patients are too good. They do not need passive mobilization (physiotherapist)

## Discussion

In this feasibility study, we show that both acute hospitalized geriatric medicine patients and their relatives accept the use of robot technology in passive mobilization. The study also shows that the newly developed mobilization robot, ROBERT®, is feasible to use within the department of geriatric medicine, although some technical and workflow-related issues affect the launch of a RCT and thereby the possibility of clinical implementation in its current design.

The overall goal in the field of rehabilitation robots is to develop implementable technologies that are simple to use and manage by therapists, clinicians, and patients. To obtain acceptance of a rehabilitation robot, a user-friendly interface is highly preferred and desirable [9]. Our feasibility study shows that ROBERT® is a simple to use robot and therefore user-friendly from the perspective of the applying physiotherapist, although it is heavy and big when moving it around from hospital room to hospital room.

Many older adults with frailty have limitations in performing active mobilization. Therefore, passive mobilization may be a relevant alternative, also as it has been found to be similarly effective for improving the functional fitness of older nursing home residents [24]. However, despite our inclusion criteria aiming at including patients in need of passive mobilization, the patients in this study improved their clinical condition quickly. Thus, the staff assessed that geriatric medicine patients are more in need of active mobilization and wished that the robot could also perform active mobilization.

However, regardless of the dubious need for passive mobilization, our study indicates that passive mobilization by the robot has a positive influence on the motivation and mood of geriatric medicine patients. A study assessing stroke patients who received early mobilization (upright and out of bed) had a less depressed mood after 7 days compared to a group receiving standard care [25]. We have no data on depression or mood, and the type of mobilization is different. However, in line with the above study, the majority of both patients and relatives in our study were very positive about the passive mobilization robot. Patients expressed that the mobilization sessions gave them motivation and that it was an activity, which they missed during the weekends.

Finally, the physiotherapist only performed the movement with the patient once and then the robot repeated these movements. In this way, the musculoskeletal system of the physiotherapist was spared. While the robot was performing the mobilization sessions, the physiotherapist could potentially use the time to perform other tasks. In this way, the available work force would be used more efficiently. However, the physiotherapist did not feel the patient could be left alone with the robot in our study because the robot lacked an emergency stop button for the patients. In addition to an emergency stop button, our feasibility study also pointed at other recommendations for technical issues to be implemented into the robot, like a display where number of repetitions can be seen, as well as a wish for a smaller and handier robot.

### Strengths and limitations

This study only focuses on the feasibility of the mobilization robot. In this way, our study does not explore the potential physical effects of the passive mobilization done by the robot. However, our study indicates that geriatric medicine patients are positive about the passive mobilization sessions by the robot and they felt it was motivating to do the sessions. Also, one patient pointed out that her legs had benefited from being activated by the robot. This finding is backed by a study on passive mobilization, which found that passive leg movements had the capacity to induce muscular activity and enhance oxygen metabolism [26]. Second, we only included few patients (*n* = 13) and relatives (*n* = 2) in this feasibility study, and our findings should therefore be interpreted with caution. However, our recruitment strategy was sufficient to recruit the number of participants required for this feasibility study and participants stated they found the study interesting and relevant. Third, due to our exclusion criteria, we excluded important geriatric patient groups such as cognitive frail patients and those with recent fractures. This may limit the external validity of our results. However, only few patients with dementia or delirium (~ 10%) were excluded, and patients with recent fractures had to be excluded due to safety. In addition, we specifically aimed at identifying patients who potentially would benefit from passive mobilization, and the majority of excluded patients were excluded since they were well mobilized already at admission. Finally, results from the interviews of the robot user should be interpreted with caution as we were only able to include one physiotherapist due to practicalities.

To our knowledge, this is the first study to pilot test and investigate the feasibility of a passive mobilization robot device in a clinical department of older geriatric medicine patients. This makes the current study important in demonstrating the feasibility of introducing newly developed robot technology in order to explore and understand the impact on patients, relatives, and health professionals. The multiple data collection methods and the feasibility framework allowed opportunities to collect various data formats.

Also, older people are often excluded from clinical trials, resulting in uncertainty about the risks and benefits of new treatments for older people [27, 28]. Clinicians and policymakers who make treatment decisions about older patients therefore often rely on research conducted primarily among middle-aged adults [29]. However, benefits shown in trials including younger people may not apply to older people [30], and trials often fail to assess the impact of interventions on the general health of older people. The evidence is thus not generalizable to typical older frail patients [28]. Therefore, we consider it to be a strength that the rehabilitation robot was tested in a real-life setting with older geriatric medicine patients.

Finally, our pilot test and feasibility study focused on analyzing data from different perspectives, to gain essential knowledge and knowhow, which are essential prior to a RCT study. The next step in the research process would be to test the effectiveness of the mobilization robot compared to conventional procedures to produce the necessary data for a power calculation in a full scale RCT including cost analysis. With our results in mind, the current robot design needs some modifications and upgrades before launching a RCT. However, due to our results and feedback, the developers have modified the robot introducing several upgrades including a stop button for the patients and the ability to perform both passive and active mobilization sessions. Whether the upgraded robot version could successfully be introduced in a geriatric ward initially requires a new pilot and feasibility study.

## Conclusion

In this pilot test and feasibility study, we show that a passive mobilization robot is feasible to use in a sample of older acutely hospitalized geriatric medicine patients. Most patients reported that they liked to do passive mobilization with the robot and that they would say yes to do it again if they got the opportunity. Furthermore, all patients felt safe during the mobilization sessions and the relatives also expressed their satisfaction. The staff performing the mobilization sessions found that the robot lived up to the expectations and expressed that it spared the physiotherapist from unhealthy and demanding working positions. However, the robot is categorized as being big and heavy when moving it around in the department and hospital rooms, as well as missing some technical functions (an emergency stop button to patients and a display). These technical and workflow-related issues, as well as the fact that the robot is not able to perform active mobilization, affect the launch of a RCT and thereby its implementation for geriatric medicine patients in its current design.

## Supplementary information

**Additional file 1: Table S1.** Interview guide for structured interviews with patients.

**Additional file 2: Table S2.** Semi-structured interview guide for physiotherapist.

## Data Availability

Please contact the corresponding author with requests. Ethical approval and associated requirements may prevent the sharing of study data.
