# Supplementary material for: Use of robot technology in passive mobilization of acute hospitalized geriatric medicine patients: a pilot test and feasibility study
Source: Pilot Feasibility Stud. 2020 Jan 6;6:1. doi: 10.1186/s40814-019-0545-z (PMC6943926; doi:10.1186/s40814-019-0545-z)
Supplement: Supplementary file 1 — Additional file 1: Table S1. Interview guide for structured interviews with patients. [file 40814_2019_545_MOESM1_ESM.docx]

| 1. Have you ever tried exercing with a training machine like this robot?  - Yes - No - Do not know/Do not remember  1. How was it to do exercise with the robot?    - Good    - Neither nor    - Not good    - Do not know 2. Were you pleased to do exercise with the robot?    - Yes    - Neither nor    - No    - Do not know 3. Did you feel safe while doing exercise with the robot?    - Yes    - Neither nor    - No    - Do not know 4. Did you experience any unpleasent when doing exercise with the robot?    - **Yes**    - **Neither nor**    - **No**    - **Do not know** 5. Did you get enough information about the robot before you started exercising?    - **Yes**    - **Neither nor**    - **No**    - **Do not know** 6. Would you say yes to do exercise with the robot if you got the opportunity again?    - **Yes**    - **Neither nor**    - **No**    - **Do not know** 7. Do you have any other comments?    - **Yes**    - **No** |
| --- |

Additional file 1: Table S1: Interview guide for structured interviews with patients
