# Supplementary material for: Use of robot technology in passive mobilization of acute hospitalized geriatric medicine patients: a pilot test and feasibility study
Source: Pilot Feasibility Stud. 2020 Jan 6;6:1. doi: 10.1186/s40814-019-0545-z (PMC6943926; doi:10.1186/s40814-019-0545-z)
Supplement: Supplementary file 2 — Additional file 2: Table S2. Semi-structured interview guide for physiotherapist. [file 40814_2019_545_MOESM2_ESM.docx]

| Themes | Interview question and additional optional prompts |
| --- | --- |
| In general | 1. How was it to use the robot for your patients at department of geriatric medicine?  - Have you ever before used a robot/machine like this robot? |
| Technical challenges and security | 1. How was it to use the robot?  - Did you think at the robot need any other technical functions? - Did you feel safe when using the robot? - Do you have any comments regarding the security of the robot? |
| Adaptation of the physical environment | 1. How was it to maneuver the robot around in the department and to the patients?  - How do you think the robot is functioning in the surroundings of the department? - How is the cleaning of robot? |
| Working environment | 1. In what way does the robot influence your working positions when doing passive mobilization? |
| Time and quality | 1. How long time does it take to get the robot ready for a mobilization session?  - How long time was a typically mobilization session? - How was the robot in relation to perferm the passive mobilization movements? - How will you compare the quality of the passive mobilization with and without the robot? |
| Patient reaction | 1. How will you characterize the patient´s perception and motivation for the robot?  - Did the patients express any pain or unpleasantness at any time when using the robot? - Do you believe that doing passive mobilization with the robot had any effect on the patients physical and mental condition? |
| Expectations and recommendation  Other issues | 1. In what way has the robot been doing in relation to you expectations? 2. Would you recommend the use the robot at a department of geriatric medicine or in any other department? 3. Is there anything else that you would like to comment on that I haven’t already asked you about? |

Additional file 2: Table S2: Semi-structured interview guide for physiotherapist
